# Engagement and Retention: Measuring Breadth and Depth of Participant Use of an Online Intervention

**DOI:** 10.2196/jmir.1430

**Published:** 2010-11-18

**Authors:** Mick P Couper, Gwen L Alexander, Nanhua Zhang, Roderick JA Little, Noel Maddy, Michael A Nowak, Jennifer B McClure, Josephine J Calvi, Sharon J Rolnick, Melanie A Stopponi, Christine Cole Johnson

**Affiliations:** ^8^Kaiser Permanente ColoradoHealth Research CenterDenverUSA; ^7^Health PartnersHealth Services Research CenterMinneapolisUSA; ^6^Kaiser Permanente GeorgiaHealth Research CenterAtlantaUSA; ^5^Group Health Research InstituteSeattleUSA; ^4^University of MichiganCenter for Health Communications ResearchAnn ArborUSA; ^3^Unversity of MichiganSchool of Public HealthAnn ArborUSA; ^2^Henry Ford Hospital and Health SystemAnn ArborUSA; ^1^University of MichiganSurvey Research CenterAnn ArborUSA

**Keywords:** Methodological studies, Internet, process metrics, tailored intervention

## Abstract

**Background:**

The Internet provides us with tools (user metrics or paradata) to evaluate how users interact with online interventions. Analysis of these paradata can lead to design improvements.

**Objective:**

The objective was to explore the qualities of online participant engagement in an online intervention. We analyzed the paradata in a randomized controlled trial of alternative versions of an online intervention designed to promote consumption of fruit and vegetables.

**Methods:**

Volunteers were randomized to 1 of 3 study arms involving several online sessions. We created 2 indirect measures of breadth and depth to measure different dimensions and dynamics of program engagement based on factor analysis of paradata measures of Web pages visited and time spent online with the intervention materials. Multiple regression was used to assess influence of engagement on retention and change in dietary intake.

**Results:**

Baseline surveys were completed by 2513 enrolled participants. Of these, 86.3% (n = 2168) completed the follow-up surveys at 3 months, 79.6% (n = 2027) at 6 months, and 79.4% (n = 1995) at 12 months. The 2 tailored intervention arms exhibited significantly more engagement than the untailored arm (*P* < .01). Breadth and depth measures of engagement were significantly associated with completion of follow-up surveys (odds ratios [OR] = 4.11 and 2.12, respectively, both *P* values < .001). The breadth measure of engagement was also significantly positively associated with a key study outcome, the mean increase in fruit and vegetable consumption (*P* < .001).

**Conclusions:**

By exploring participants’ exposures to online interventions, paradata are valuable in explaining the effects of tailoring in increasing participant engagement in the intervention. Controlling for intervention arm, greater engagement is also associated with retention of participants and positive change in a key outcome of the intervention, dietary change. This paper demonstrates the utility of paradata capture and analysis for evaluating online health interventions.

**Trial Registration:**

NCT00169312; http://clinicaltrials.gov/ct2/show/NCT00169312 (Archived by WebCite at http://www.webcitation.org/5u8sSr0Ty)

## Introduction

The major advantages of online interventions lie in their ability to reach large numbers of potential clients with very complex individually tailored designs and with relatively low cost [1,2]. One of the disadvantages of online interventions is the lack of “stickiness”—the ability to attract and retain Internet visitors—relative to other modes of contact [3,4]. Providing people access to a website is no guarantee that they will use it. A key concern is with lack of engagement [5] in online interventions, leading to dropout from the study and loss to follow-up or to dampening of the treatment effect [6-8]. However, unlike other media for health interventions (especially those not involving direct human contact), logs of access and use of online interventions can give researchers insight into what people are doing and when they are doing it. Such interventions provide tools to learn more about participant engagement and, further, how that relates to retention and intervention outcomes. This information can be used to understand the dynamics of engagement and can lead to design changes to improve the retention and engagement of online health behavior interventions.

This paper focuses on what is variously called dosage [9], exposure [10,11], adherence [12], or engagement [5]. As Danaher et al [11] note, “a key ingredient in determining the impact of any Web-based behavior change program is the extent to which participants are exposed to the program.” We use the paradata from an online intervention to explore the level of engagement and factors associated with user engagement in the intervention. Paradata are auxiliary data that capture details about the *process* of interaction with the online intervention [13]. Some paradata are captured as a matter of course when users connect to a website. These user metrics contain information on the user’s browser, connection speed, and other details about user behaviors. Other types of paradata must be captured as an explicit part of the design of the site, using a variety of tools such as cookies, Web bugs, and session identifiers. These can include information on which pages are visited, when and how often, and for how long. This kind of information can provide insight into what people are spending time on and, more importantly, what they are ignoring and missing. Paradata are widely used in Web survey settings to learn more about respondent behavior [14-18], but have not been widely used in online health interventions, with some exceptions [6,11,19].

The goal of this paper was to use paradata to explore engagement in a randomized controlled trial (RCT) of an online intervention with several different arms. Specifically, we examined both breadth and depth of engagement defined in new measures built from paradata. We then explored how engagement was related to retention in the study, as measured by completion of the follow-up surveys. Finally, we addressed the relationship between engagement and key outcomes of the trial. Our expectation was that tailored interventions would result in greater engagement in the online material, leading to lower attrition in the intervention and improved outcomes. This paper provides a starting point to identify areas where online intervention design improvements may be required and, ultimately, may give us clues as to why a particular intervention may be more or less successful.

## Methods

Data for this study came from the Making Effective Nutritional Choices for Cancer Prevention (MENU) study (Trial Registration: ClinicalTrials.gov NCT00169312), a randomized trial conducted in conjunction with the Cancer Research Network (CRN). The CRN is a consortium of 14 research organizations affiliated with nonprofit integrated health care delivery systems and the National Cancer Institute (NCI) [20,21]. The MENU study tested a randomized longitudinal intervention utilizing an interactive website to promote greater intake of fruit and vegetables [22]. In total, 5 of the CRN affiliated health care delivery systems in their headquarter cities—Group Health Cooperative in Seattle, Kaiser Permanente Colorado in Denver, HealthPartners in Minneapolis, Henry Ford Health System in Detroit, and Kaiser Permanente Georgia in Atlanta—collaborated with the University of Michigan’s Center for Health Communications Research, which provided Web design and support for the MENU study. The online intervention offered 4 core education sessions phased over a 4-month period with 4 assessment surveys at baseline, 3-, 6-, and 12-months post enrollment. Sessions included motivation support, information, and “how to” behavioral strategies, and offered supplemental “special features,” a bank of 300 fruit and vegetable-based recipes, plus food preparation videos. All enrollment processes and assessment surveys were completed online. Participants were enrolled between September 2005 and March 2006. All protocols were approved by the institutional review boards of the participating institutions.

### Participants

Study subjects, aged 21 to 65 years, were randomly selected and recruited from the administrative databases of the 5 participating health care systems. Selection was limited to those members who had at least one-year enrollment in the respective health plan and had no record (according to diagnostic codes) of existing health conditions that might be negatively affected by increasing dietary fruit and vegetables. Equal numbers of men and women were selected, and 3 sites over-sampled minority racial/ethnic groups (African American or Hispanic) to enhance diversity in enrollment. Access to the Internet for personal use and use of a working email account, assessed during the study’s online eligibility survey, was also required for enrollment.

Of the 28,460 members mailed invitations to participate in the study, 4270 (15%) visited the website and 2540 (8.9% of those invited or 59.5% of those visiting the website) enrolled. Analysis following the 12-month survey identified 27 participants who reported inconsistencies in birth date and gender, suggesting different people may have completed the follow-up surveys. These cases were dropped from all analyses, leaving a final count of 2513 participants for analysis (Table 1). Further information on the enrollees is provided in Stopponi et al [23]. Of the 2513 enrollees, the average age was 46.3 years, 69% were women, 66% were white or other non-Hispanic, 24% were African American, and 8% were Hispanic; 51% of enrollees had a college education or higher.

### Recruitment Procedures

Participants were recruited with a single mailed invitation letter using health system stationery. The letter described eligibility criteria and included the Web address and a unique sign-on code which could be used to access more information about the study online. Also included were a US $2 bill preenrollment incentive and the promise of US $20 for completing each of the 3 follow-up surveys during the 12-month follow-up period [24]. After logging in online, individuals were asked for permission to proceed through the eligibility screening questions (9 to 12 questions, depending on personal tailoring). If eligible, individuals were given information about the study (the information was displayed and distributed across 8 consecutive Web pages) and were asked to provide informed consent. Those who consented were asked to provide their personal contact information (ie, phone, email, and mailing address). Email addresses were verified, and consenting individuals were asked to complete the first (baseline) survey after which they were randomized to a study arm. Participants were encouraged to complete the enrollment process in one sitting but could complete it in more than one session if necessary.

### Intervention

Enrollees were randomized to 3 experimental arms receiving Web sessions that were (1) untailored, (2) tailored, or (3) tailored with email support which utilized a human online behavioral intervention (HOBI) consisting of behavior change counseling. Randomization was assigned by study site, gender, and stage of change with eating fruit and vegetables. Tailored Web sessions were based on health risk information and motivations for change obtained from baseline or 3-month post surveys. All materials were provided in English only.

An initial online welcome letter showed the participant’s current status of reported fruit and vegetable servings compared with recommended intakes [25] and explained the sequence of the 4 core Web sessions. Web sessions were similar in design and educational content, which was focused on nutritional information and cognitive and behavioral support to eat more fruits and vegetables. The welcome session was available immediately following the baseline assessment, and subsequent intervention sessions were made available at 1-, 3-, 13-, and 15-weeks postenrollment. An automated process sent emails when new content was available for review. All materials were available, once presented, through the end of the 12-month study period.

The MENU tailored Web program included content and suggestions matched to each person’s gender, needs, characteristics, dietary preferences, and interests. Behavioral sessions were tailored to each person’s stage of change and were designed to increase participants’ motivation and self-efficacy for buying, preparing, and eating fruits and vegetables. Tailored web sessions also contained tailored video and audio files designed to reinforce behavioral advice featuring videos of food preparation by Graham Kerr, a well-known, health-conscious chef. Additionally, persons in the tailored arms were able to access an expert-tailored menu, which was generated based on their fruit and vegetable preferences, dietary restrictions, and other preferences.

In addition to the tailored program, participants in Arm 3 were offered corresponding email counseling support sessions. Each counseling session was initiated by a study counselor within a week after each Web session was first visited. Counselors provided additional support for dietary change, following the therapeutic principles outlined in motivational interviewing [26,27]. Counselors responded to any request for strategies or for nutrition information with a referral to the MENU website. A maximum of 4 unique email discussions corresponding to each of the 4 Web sessions were initiated by the counselor when the sessions were accessed. Each email discussion was limited to 4 “back and forth” exchanges.

### Special Features

In addition to the sessions, participants could access “special features,” which were short, optional, and individually accessed clusters of Web pages that appeared periodically on the intervention website and which presented tips and other additional information in a pop-up window. Like sessions, notice of each feature’s availability was automatically delivered a fixed number of days after enrollment. Examples of special features included recipes developed by Graham Kerr, a dietary intake goal-setting tool, tips for eating out, food safety and storage, fun with fruit and vegetables, and nutritional similarities of fresh, frozen, and canned foods (for details, see [22]). Participants reporting children in their household received a special feature on encouraging kids to eat fruit and vegetables, while those reporting no children were given a special feature on preparing quick and healthy foods. A total of 17 unique special features were offered, but only 16 were available for any one participant since one was tailored to parental status. Once available and accessed, special features could be revisited. We tracked the total number of times, if any, that participants accessed each special feature.

### Data Collection Procedures

The Web protocol for all data collection surveys was similar. Participants were asked to report fruit and vegetable intake at baseline, 3, 6 and 12 months, using one or both of two fruit and vegetables screeners. The first, used at baseline and 12 months, is based on a 16-item measure of fruit and vegetable servings, adapted from the NCI 19-item fruit and vegetable food frequency questionnaire [28]. The second, used at all 4 assessment time points, is based on a 2-item measure assessing total servings of fruit and vegetables on a typical day [29]. Also included in the baseline survey were questions about intrinsic and extrinsic motivations, barriers to eating fruit and vegetables, and confidence about making dietary changes. Intrinsic and extrinsic motivation for eating fruit and vegetables were assessed using a 14-item subset of the Treatment Self-Regulation Questionnaire (TSRQ) measure developed by Williams and Deci [30] and modified to apply to fruit and vegetable intake by Resnicow et al [31]. Living status and demographics were also assessed.

**Table 1:** Baseline description of the enrolled subjects by study arm

			Study Arm
Variable		Total (n = 2513)	Arm 1 Control (n = 836)	Arm 2 Tailored (n = 839)	Arm 3 Tailored + HOBI (n = 838)
Age (years), mean (SD) median		46.3 (10.8) 48.0	46.1 (10.6) 47.0	46.5 (10.8) 48.0	46.4 (10.9) 47.0
Female, n, %		1729 (69)	576 (69)	577 (69)	576 (69)
African American, n, %		585 (24)	192 (23)	196 (24)	197 (24)
Hispanic, n, %		192 (8)	69 (8)	66 (8)	57 (7)
Married/with partner, n, %		1805 (72)	595 (72)	602 (72)	609 (73)
High school education or less, n, %		217 (9)	76 (9)	70 (8)	71 (9)
Associate or some college, n, %		1023 (41)	334 (40)	352 (42)	337 (40)
College degree, n, %		659 (26)	219 (26)	232 (28)	208 (25)
Post bachelor’s education, n, %		607 (24)	205 (25)	183 (22)	219 (26)
**Fruit consumption, stage of change**					
	Precontemplator stage, n, %	49 (2)	17 (2)	14 (2)	18 (2)
	Contemplator stage, n, %	1247 (50)	412 (49)	421 (50)	414 (49)
	Preparation stage, n, %	511 (20)	164 (20)	175 (21)	172 (21)
	Action stage, n, %	170 (7)	54 (6)	61 (7)	55 (7)
	Maintenance stage, n, %	533 (21)	189 (23)	166 (20)	178 (21)
**Vegetable consumption, stage of change**					
	Precontemplator stage, n, %	40 (2)	11 (1)	17 (2)	12 (1)
	Contemplator stage, n, %	1547 (62)	519 (62)	523 (62)	505 (60)
	Preparation stage, n, %	389 (15)	128 (15)	124 (15)	137 (16)
	Action stage, n, %	104 (4)	35 (4)	35 (4)	34 (4)
	Maintenance stage (%)	430 (17)	143 (17)	138 (16)	149 (18)
	Fruits and vegetables/day, 16-item measure of servings: mean^a^ (SD) median	4.4 (2.8) 3.8	4.6 (3.0) 3.9	4.2 (2.7) 3.6	4.5 (2.7) 4.0
	Fruits and vegetables/day, 2-item measure of servings: mean (SD) median	3.3 (1.58) 3.0	3.3 (1.57) 3.0	3.2 (1.57) 3.0	3.4 (1.59) 3.0

^a^ Using the Kruskal-Wallis test, the means by arms were statistically significantly different at *P* = .049.

### Measures

#### Outcome Measures

We examined the role of engagement in minimizing attrition or maximizing retention in the study. We defined retention as completion of the follow-up surveys at 3-, 6-, and 12-months after baseline.

We also examined two key substantive outcomes measured as change in mean fruit and vegetable consumption from baseline to 12-month follow-up. In both cases, a positive score indicated an increase in consumption. The 2 measures were correlated (*r* = .60), with the shorter 2-item measure having had a higher 12-month completion rate.

The baseline survey included 70 questions and took an average of 25 minutes to complete. The 3-month follow-up survey included 32 questions, taking an average of 13 minutes to complete; the 6-month survey included 30 questions, taking an average of 13 minutes to complete; and the 12-month survey included 80 questions, taking an average of 29 minutes to complete. A reminder letter was mailed to all enrollees a week prior to each survey due date, and an email reminder was sent to all enrollees on each survey due date. A series of 5 automated reminder emails were sent to anyone who had not completed the survey every 3 or 4 days after the due date. For the 3-month survey, phone call reminders were initiated in the final 5 days of the online completion “window” during which callers offered enrollees reminders to do the survey and the opportunity to complete the survey by phone. Nearly all of the assessments (> 96%) were completed online. Overall, 86.3% of baseline participants completed assessments at 3 months, 79.6% at 6 months, and 79.4% at 12 months with no significant differences by intervention arm.

#### Paradata Measures

The engagement measures were obtained using server-side paradata. For confidentiality reasons we did not embed JavaScript code in the Web pages to capture client-side paradata [17]. We used the time stamps from the following 5 primary actions: (1) logging in to the website; (2) initiation of any of the 4 online surveys; (3) completion of any of the online surveys; (4) loading the first page of any of the 4 core Web sessions; and (5) loading the first page of any 1 of the 17 special features. The website automatically logged out the participant after 30 minutes if there was no new participant-generated activity. If logged out, the participant would need to repeat the log-in process, generating another log-in event.

#### Total Sessions

The MENU program consisted of 4 sessions, each made available at different time points: 3 days after baseline, 21 days after baseline, 3 days after the 3-month survey, and 21 days after the 3-month survey. Once new content was available, the user was automatically presented with the current new session at log-in. A bank of nearly 300 recipes and a goal-setting feature were available as optional elements throughout the study. All previous sessions remained available in a navigation bar at the top of the Web page. Participants could thus view up to 4 unique informational sessions by the end of the intervention program; however, the total count of sessions accessed could be higher if a session was viewed more than once.

#### Unique Sessions

The measure “unique sessions” was simply a count of the number of offered informational Web sessions visited at least once, with the maximum being 4.

#### Time Online

To approximate the total time spent interacting with the website over the course of the study, we attributed the elapsed time between 2 time-stamped events to the action that generated the first of the events. These elapsed times were then accumulated across the various actions to give total elapsed times for each type of action done on the website. These accumulated times may have been slightly lower than the time actually spent on the site since we did not capture how long the participant spent reading the previously accessed Web session or special feature.

#### Engagement

We focused on the 4 measures of engagement captured through the website paradata and described above: total session accesses, unique session accesses, total special feature accesses, and total time on the website (excluding time spent completing the surveys) (see Table 2). Given that all 4 measures are related, we sought to create more parsimonious summary measures of engagement. The 4 measures were subjected to a principal components analysis (PCA). The first 2 principal components accounted for 90% of the total variation in program usage between study participants, with the first accounting for 73% and the second for 17% of the total variation. Based on this, the following 2 summary measures were created:

- BREADTH is a summary measure of access to all activity on the website. It is composed of the sum of the 4 measures in Table 2, standardized by dividing by their standard deviations to compensate for the differences in scales. BREADTH approximates the first principal component from the PCA.
- DEPTH is a summary measure of how deeply individuals engaged in the online material, for a given level of overall Web activity. NON_SURVEY_MINS (total minutes spent excluding survey completion) and SF_TOT (total number of special feature accesses) loaded positively on the second principal component, while SESS_UNIQ (number of unique session accesses) loaded negatively, with the loading of SESS_TOT (total number of session accesses) close to 0. The measure of DEPTH is thus obtained as the sum of the average (standardized) total of accessed special feature sessions (SF_TOT) and standardized nonsurvey minutes spent online (NON_SURVEY_MINS), minus twice the total number (standardized) of unique sessions accessed (SESS_UNIQ). The more special features a person accessed, and the longer they spent on the website relative to the number of different sessions they saw, the higher the value of DEPTH. DEPTH approximates the second component from the PCA.

Using the factor loadings from the PCA yielded similar results to those using the methods described above. The measures of BREADTH and DEPTH were again standardized (mean 0, SD 1) for further analyses. The two measures were slightly positively correlated, *r* = .12. Based on the PCA, we named these 2 measures to indicate that they measured different aspects of engagement.

In the multivariate models, we controlled for a number of additional variables measured at baseline. Fruit and vegetable consumption was based on the sum of 2 single measures and collapsed into low (less than 2 servings per day), medium (2 to 4 servings per day), and high (5 or more servings per day) consumption.

### Statistical Analysis

We focused on several outcomes of interest. First, utilizing our 2 newly derived indicators of the depth and breadth of engagement based on PCA, we explored the correlates of these engagement indicators from the baseline survey, using ordinary least squares (OLS) regression. Next, we examined completion of the follow-up surveys using both the baseline measures and the 2 new engagement indicators as predictors. These analyses used generalized estimating equations (GEE), reflecting the within-subject correlation across outcomes. A likelihood ratio chi-square was used to test whether the addition of the 2 engagement indicators improved the model fit. Finally, we examined 2 key outcome measures (fruit and vegetable consumption at 12 months) to explore how engagement may mediate the effect of the intervention on outcomes. The models again used OLS regression. Statistical analyses were done using SAS 9.1.3(SAS Institute Inc, Cary, NC, USA).

## Results

The 4 component indicators of engagement are presented in Table 2. On average, participants visited the sessions a total of 10.6 times across the course of the intervention; no differences were identified by study arm. In terms of unique sessions, not all sessions were seen by all participants, with an average of 3.1 sessions visited, overall. Of all participants, 5.1% (128/2513) of participants did not visit any of the 4 sessions. Just over half (1410/2513, 56.1%) visited all 4 unique sessions; this did not vary by intervention arm. Similarly, on average, participants visited special features an average of 11.1 times, with 13.7% (344/2513) not visiting special features at all.

**Table 2:** Descriptive statistics on component engagement measures (n=2513)

Variable	Mean	SD	Median
Total session accesses (SESS_TOT)	10.64	7.14	9
Unique session accesses (SESS_UNIQ)	3.14	1.20	4
Total special feature accesses (SF_TOT)	11.13	10.79	8
Total time excluding survey completion (NON_SURVEY_MINS)	42.16	42.93	29.55

The mean number of special feature accesses (8.3 for arm 1, 10.2 for arm 2, 10.3 for arm 3) and mean total minutes devoted to the intervention website (32.3 for arm 1, 44.1 for arm 2, 46.7 for arm 3) differed significantly by arm (F_2,2512_ = 9.57, *P* < .001 and F_2,2512_ = 27.04, *P* < .001, respectively). Levels of engagement with accessing special features and time spent on the Web intervention were lower in the untailored arm for both measures, with higher and nearly equivalent levels observed when comparing the 2 tailored arms.

### Correlates of Engagement

We regressed the standardized measures of depth and breadth, in turn, on a series of sociodemographic and related behavioral variables at baseline, using OLS regression (SAS 9.1.3 PROC GLM, SAS Institute Inc, Cary, NC, USA). These models are presented in Table 3.

**Table 3:** Models of standardized breadth and depth regressed on common demographic/baseline variables

Predictors		Breadth		Depth	
		Coefficient	(SE)	Coefficient	(SE)
**Arm**					
	Arm 1: Untailored	---	---	---	---
	Arm 2: Tailored	0.114 ^b^	(0.047)	0.234 ^b^	(0.049)
	Arm 3: Tailored with HOBI	0.141 ^b^	(0.047)	0.305 ^b^	(0.049)
**Female versus male**		0.407 ^b^	(0.044)	0.087	(0.046)
**Age**					
	< 29	-0.475 ^b^	(0.101)	-0.428 ^b^	(0.104)
	29-38	-0.437 ^b^	(0.081)	-0.315 ^b^	(0.084)
	39-48	-0.230 ^b^	(0.074)	-0.262 ^b^	(0.077)
	49-58	-0.047	(0.068)	-0.182 ^b^	(0.070)
	59+	---	---	---	---
**Race**					
	White	---	---	---	---
	Black	-0.045	(0.050)	0.098	(0.052)
	Other	-0.034	(0.071)	-0.110	(0.073)
	Hispanic versus non Hispanic	-0.127	(0.086)	0.094	(0.088)
**Education**					
	High school or less^c^	---	---	---	---
	Some college	0.110	(0.059)	-0.150 ^a^	(0.061)
	College graduate	0.106	(0.063)	-0.137 ^a^	(0.065)
	Postgraduate	-0.026	(0.064)	-0.265 ^b^	(0.067)
**One or more children in home versus none**		-0.167 ^b^	(0.046)	-0.062	(0.047)
**Marital status**					
	Never married	---	---	---	---
	Formerly married	-0.048	(0.079)	0.007	(0.082)
	Married/living with partner	-0.015	(0.066)	0.071	(0.068)
**Self-reported health**					
	Poor to good	-0.050	(0.042)	0.063	(0.044)
	Very good to excellent	---	---	---	---
**Fruit and vegetable consumption**					
	Low	-0.047	(0.063)	0.101	(0.065)
	Medium	---	---	---	---
	High	0.031	(0.052)	-0.054	(0.054)
**Comfort using Internet**					
	Low	-0.126 ^a^	(0.057)	-0.071	(0.059)
	Medium	---	---	---	---
	High	-0.078	(0.048)	-0.069	(0.050)

**Motivation to eat more fruit**					
	Low	-0.126 ^a^	(0.057)	0.018	(0.056)
	Medium	---	---	---	---
	High	-0.078	(0.063)	0.015	(0.060)
**Motivation to eat more vegetables**					
	Low	0.021	(0.054)	-0.027	(0.056)
	Medium	---	---	---	---
	High	-0.074	(0.058)	-0.023	(0.060)
**Physical activity level**					
	Inactive	0.197 ^a^	(0.094)	0.182	(0.098)
	Low activity	0.163 ^a^	(0.066)	0.047	(0.068)
	Somewhat active	0.100	(0.059)	0.065	(0.061)
	Very active	---	---	---	---
**Motivation**					
	Intrinsic motivation^d^	0.074 ^b^	(0.021)	0.026	(0.026)
	Extrinsic motivation^e^	-0.047 ^b^	(0.014)	-0.017	(0.014)
**Model fit**					
	Constant	-0.287	(0.195)	-0.259	0.202
	Observations	2461		2461	
	R^2^	.108		.053	

^a^ *P* < .05

^b^ *P* <.01

^c^ Category includes those with vocational or technical training.

^d^ Intrinsic motivation measures personal importance or internal drive to do a behavior. Examples are: “I have a strong value for eating healthy” and “I want to take responsibility for my own health.”

^e^ Extrinsic motivation measures perceived outside influences on behavior. Examples are: “Others would be upset with me if I didn’t (eat more fruits and vegetables)” and “It is easier to do what I am told.”

Together these baseline measures explained a modest proportion of variation in the breadth (*R* *2* = .108) and depth (*R* *2* = .053) of engagement in the online materials. In terms of experimental conditions, those exposed to either of the 2 tailored conditions exhibited significantly more overall online activity than those exposed to the untailored materials. Women had significantly higher levels of breadth (exposure to a variety of items in the intervention) than men, but depth (more time dedicated to the intervention materials) did not differ by gender. Age was significantly associated with both engagement measures, with lower levels of engagement exhibited by younger participants. Race and ethnicity were not associated with differences in engagement. Education was significantly associated with the depth measure, with lower engagement (eg, less time online, fewer special feature accesses) by those with higher levels of education. The presence of children in the home was negatively associated with breadth of engagement but not with depth, and marital status showed no association with either breadth or depth.

Few of the baseline measures showed significant associations with the measures of engagement in the program. Low comfort using the Internet was significantly related to lower breadth, or amount of the website seen. Those with low motivation to eat fruit upon enrollment exhibited slightly lower breadth of engagement, but those who were less physically active showed higher levels. Intrinsic motivation was positively associated with depth, while extrinsic motivation was negatively associated with depth.

### Predictors of Survey Completion

In the second step, we used the standardized breadth and depth measures of engagement along with all of the baseline measures included in Table 2 to predict completion of the follow-up surveys at 3-, 6-, and 12-months after baseline. Our expectation was that those who were less engaged in the online material would be less likely to complete the follow-up surveys.

We used a generalized estimating equation (GEE) in SAS 9.1.3 PROC GENMOD to model survey completion, reflecting the within-subject correlation across outcomes [32]. The odds ratios and 95% confidence intervals for survey completion are presented in Table 4.

**Table 4:** Model of survey completion at 3-, 6-, and 12-months

Predictors		Odds Ratio	95% Confidence Interval
**Follow-up survey**			
	3-month	1.0	
	6-month	0.48 ^b^	(0.42-0.56)
	12-month	0.48 ^b^	(0.41-0.55)
**Arm**			
	Arm 1: Untailored	1.0	
	Arm 2: Tailored	0.82	(0.64-1.06)
	Arm 3: Tailored with HOBI	0.79	(0.61-1.02)
**Female versus male**		1.07	(0.85-1.35)
**Age**			
	< 29	1.06	(0.63-1.81)
	29-38	1.1	(0.71-1.69)
	39-48	1.03	(0.70-1.54)
	49-58	0.92	(0.63-1.34)
	59+	1.0	
**Race**			
	White	1.0	
	Black	0.85	(0.66-1.09)
	Other	0.82	(0.57-1.17)
	Hispanic	0.63 ^a^	(0.41-0.97)
**Education**			
	High school or less	1.0	
	Some college	0.86	(0.64-1.15)
	College graduate	1.02	(0.74-1.41)
	Postgraduate	1.32	(0.94-1.85)
**One or more children in home versus none**		1.00	(0.79-1.27)
**Marital status**			
	Married	1.0	
	Formerly married	0.99	(0.66-1.50)
	Never married	1.24	(0.87-1.77)
**Self-reported health**			
	Poor to good	0.77 ^a^	(0.62-0.96)
	Very good to excellent	1.0	
**Fruit and vegetable consumption**			
	Low	0.77	(0.58-1.04)
	Medium	1.00	
	High	1.00	(0.74-1.34)
**Comfort using Internet**			
	Low	0.97	(0.72-1.30)
	Medium	1.0	
	High	1.06	(0.82-1.36)
**Motivation to eat more fruit**			
	Low	0.97	(0.73-1.29)
	Medium	1.0	
	High	1.17	(0.86-1.59)
**Motivation to eat more vegetables**			
	Low	1.29	(0.96-1.73)
	Medium	1.0	
	High	0.73 ^a^	(0.54-0.99)
**Activity level**			
	Inactive	0.68	(0.43-1.09)
	Low activity	0.89	(0.62-1.29)
	Somewhat active	0.91	(0.65-1.26)
	Very active	1.0	
**Motivation**			
	Intrinsic motivation (see Table 3)	0.98	(0.86-1.12)
	Extrinsic motivation (see Table 3)	0.96	(0.90-1.03)
			
Breadth		4.11 ^b^	(3.61-4.69)
Depth		2.12 ^b^	(1.89-2.38)
Constant		22.55 ^b^	(7.95-63.92)
**Model fit**		
	Observations	7383	
	Max-rescaled *R2*	.32	

^a^ *P* < .05

^b^ *P* < .01

From the model, we can see a significant drop-off in completion from the 3-month follow-up to the 6-month follow-up, but not from the 6-month to the 12-month. What is striking from Table 4 is that few of the baseline measures—with the exception of Hispanic origin and motivation to eat more vegetables—are predictive of survey completion following the start of the intervention.

However, our main focus was on the role of the 2 engagement measures. Both were significantly and strongly associated with survey completion. The likelihood ratio (LR) chi-square test of the addition of these two variables to the model was significant (LR χ^2^_2_ = 1005.8, *P* < .001). The Nagelkerke [33] adjusted generalized coefficient of determination (or max-rescaled *R* *2*), which is analogous to the multiple *R* *2* in linear regression, increased from .05 from the model without these two variables to .32 for the model including BREADTH and DEPTH. Thus, the level of engagement in the online materials was highly associated with completion of the follow-up surveys or attrition in the study.

### Predictors of the Key Outcomes

Finally we added BREADTH and DEPTH to a model regressing 2 key fruit and vegetable intake outcome variables on the baseline measures included in the models in Table 4 to examine whether the measures of engagement added to the explanation of the study outcomes. Given that our focus was on the role of engagement, we do not present the full models. In the 2 regression models predicting change in fruit and vegetable consumption, the engagement measures added significantly to the explained variance (F_2,1722_ = 22.08 for the 16-item measure and F_2,1945_ = 29.9 for the 2-item measure). Examining the individual coefficients, BREADTH was statistically significant (*P* < .001) in both models, while DEPTH failed to reach significance (*P* = .83 and *P* = .92 respectively), although both coefficients were in the expected direction. We tested the interactions of the engagement measures with study arm for both outcomes, and none of them was statistically significant. We thus found a main effect of engagement (specifically, BREADTH) on change in fruit and vegetable consumption, with greater breadth of engagement associated with greater (positive) change in fruit and vegetable consumption.

## Discussion

This paper focused on the use of paradata to measure the process of engagement in an online intervention aimed at increasing fruit and vegetable consumption. These data, collected throughout delivery of these online materials, reveal what pages of informational sessions are visited and the frequency and duration of the visit, but they do not reveal *why* a participant may find a particular element of the online content engaging. Paradata are thus indirect measures of engagement. We learned several things from the analysis of paradata.

### Principal Results

First, those in the 2 tailored intervention arms showed higher levels of engagement—as indicated by the 2 composite measures, BREADTH and DEPTH of engagement—than those in the untailored arm. This suggests that the tailoring is responsible for participants’ increased use of the program materials. Variation in engagement by demographic characteristics may indicate groups’ differing levels of interest in the program or online materials. Whether this is a reaction to the intervention content or a reflection of preexisting differences in interest that were not captured by our baseline measures is unclear.

Second, the engagement indicators were significant correlates of attrition from the intervention. This suggests that the more participants are engaged with the online materials, the more likely they are to complete the follow-up surveys. This is a key finding, as discovering mechanisms that promote collection of more complete outcome measures is essential to research studies.

Finally, engagement was also significantly associated with the key behavioral outcomes of the study: changes in fruit and vegetable consumption. Those who spent more time on the website, who visited a greater number of pages, and who visited the site more often, as captured by the composite measure of breadth of engagement, showed significantly greater gains in fruit and vegetable consumption from baseline to 12-month follow-up than did those who exhibited less engagement. This finding provides further empirical evidence that “dose matters” in Web-based interventions. [9]

### Strengths and Limitations

Key strengths include the large number of participants and the racial/ethnically diverse sample of relatively healthy adults from 5 geographic regions, providing a large number for analyses by subgroup. The relatively high response rates for the follow-up surveys permitted analysis of baseline and process variables to understand change in eating behaviors. Paradata measures were collected with date and time stamps over the 12-month study duration, which permitted the creation of duration and frequency variables and quantified the time lapse between website visits.

Limitations include the requirement that participation eligibility include both access to the Internet and an active email account, so findings may not generalize to all Internet users. We also were limited in the detail of the paradata we collected, as we were limited in measuring interruptions or distraction time during a Web encounter. This may have influenced our ability to distinguish between “sessions” and “visits” and did not provide details on what participants did within website sessions. Further, the incentives paid for participation, which were equivalent across intervention arms, and the effort taken to retain participants, relying mainly on automated email and single mailed reminders, may limit generalizability to other online interventions regarding the levels of engagement.

This paper demonstrates the usefulness of paradata in providing insight into the process by which an online intervention may affect outcomes. Such data are useful in identifying the “active ingredients” in a tailored intervention, that is, what works and what does not. Paradata could also be used to improve the design of online health interventions and websites, whether tailored or not, by identifying such components as which features visitors use, what pages they visit and revisit, and how long they spend on various parts of the site. This information could be used, in combination with other methods such as debriefing questionnaires or usability tests, to identify areas for program improvement, either in content or in navigation. We used a limited set of paradata captured in this online intervention. It is relatively easy to embed richer measures in health websites to provide more insight into what users are doing when they visit such sites. As online interventions increase in utilization and extend accessibility to various populations, we urge the collection and reporting of analysis of expanded paradata measures to improve the design and effectiveness of online health interventions.

## Abbreviations

CRN: Cancer Research Network
GEE: generalized estimating equations
HOBI: human online behavioral intervention
LR: likelihood ratio
MENU: Making Effective Nutritional Choices for Cancer Prevention study
NCI: National Cancer Institute
NON_SURVEY_MINS: total time excluding survey completion
OLS: ordinary least squares regression
PCA: principal components analysis
RCT: randomized controlled trial
SESS_TOT: total session accesses
SESS_UNIQ: unique session accesses
SF_TOT: total special features accesses
TSRQ: Treatment Self-Regulation Questionnaire
